# Emerging biomarkers for early diagnosis of pancreatic cancer

**DOI:** 10.1016/j.ebiom.2022.104064

**Published:** 2022-05-11

**Authors:** eBioMedicine

Pancreatic ductal adenocarcinoma (PDAC) is one of the most lethal types of human cancer. Over the past 5 years, the 5-year survival rate for people with PDAC has increased incrementally, from 6% to 10%, largely owing to improved therapeutics. Surgery remains the only curative therapy. However, PDAC can quickly advance to the metastatic stage. For up to 85% of patients with PDAC, their tumours are not surgically resectable at the point of diagnosis, making therapeutic options for these patients limited. Non-invasive biomarkers have been widely studied for PDAC diagnosis in blood and urine samples, but only carbohydrate antigen 19-9 (CA19-9) has received approval from the US Food and Drug Administration. Unfortunately, the sensitivity and specificity of CA19-9 are inadequate for PDAC diagnosis, and its clinical use is restricted to monitoring treatment response and recurrence in patients who are already diagnosed with PDAC. There is an urgent clinical need for better biomarkers and tools for the early diagnosis of PDAC.

In a study published in *eBioMedicine* in January, 2022, researchers from Hospital del Mar Medical Research Institute (Spain) reported soluble AXL (sAXL) in plasma samples as a novel non-invasive biomarker for PDAC diagnosis at the initial stages of the disease. The authors of the study showed that plasma sAXL levels could discriminate PDAC from chronic pancreatitis, with better performance than the CA19-9 biomarker (Area under the ROC Curve (AUC) 0·827 *vs* 0·673), and blood detection of both sAXL and CA19-9 panel by ELISA improved accuracy of diagnosis of PDAC (89·9% sensitivity and 100% specificity). In addition to protein biomarkers, RNAs in extracellular vesicles from plasma samples could be a putative diagnostic biomarker for PDAC. In their study published in *Gut* in 2020, Shenglin Huang and colleagues from Fudan University (China) conducted long RNA (exLR) profiling on extracellular vesicles in patients with PDAC, patients with chronic pancreatitis, as well as healthy participants and developed a diagnostic signature that comprised eight exLRs for early detection of PDAC. This diagnostic signature showed a higher ability to distinguish PDAC from chronic pancreatitis (AUC 0·931) than CA19-9 (0·873).

As with blood, urine is another convenient biofluid for the detection of pancreatic cancer at the resectable stage. Both protein and RNA biomarkers in urine samples have been widely reported. Patterns of volatile organic compounds in urine specimens, detected by ion mobility spectrometry-based methods, have the ability to distinguish healthy individuals from patients with PDAC. In their study published in *British Journal of Cancer* in 2020, researchers from Queen Mary University of London (UK) reported a urinary biomarker panel (REG1B, LYVE1, and TFF1) and constructed a biomarker-based risk score, termed as the PancRISK for early detection of PDAC, which is currently being validated in a multicentre, prospective, observational study UroPanc (NCT04449406).

Studies have shown that microbiota might affect the malignant phenotype and prognosis of pancreatic cancer, and the association of microbiome with pancreatic cancer has been widely reported. In a recent study published in *Gut* in December, 2021, researchers from Spain and Germany explored the potential of faecal and salivary microbiota as diagnostic biomarkers for PDAC. They showed that faecal metagenomic classifiers had much better performance than saliva-based classifiers and could identify patients with PDAC with an AUC score of up to 0·84 based on a set of 27 microbial species, with consistent accuracy across early and late disease stages. When combined with serum levels of CA19-9, the diagnostic performance further improved up to an AUC score of 0·94, indicating the potential for non-invasive, robust, and specific faecal microbiota-based early diagnosis for PDAC.

Early diagnosis is essential and challenging for patients with PDAC. Despite its high mortality rate, the prevalence of PDAC is very low, with an incidence of 8–12 cases per 100 000 people per year, and a 1·3% lifetime risk for the disease. This low prevalence means that there could be unacceptably high rates of false-positive results in population-based screening, which could lead to unnecessary biopsies for a definitive diagnosis. However, screening individuals in high-risk groups can increase accuracy and reduce false-positive results. Therefore, a 3-step approach (known as the DEF approach, owing to the first letters of each step) has been agreed upon in the field: (1) define what constitutes a high-risk group, (2) enrich the high-risk group further to define a very high-risk group and, (3) find the lesion in the very high-risk group.

Following the DEF approach, in their very recent *eBioMedicine* paper published in January, 2022, a collaborative team led by Eithne Costello from University of Liverpool (UK) reported a method that can enrich the very high-risk subpopulation in patients with new-onset type 2 diabetes, which represents the largest high-risk group for PDAC. A subgroup of individuals with new-onset type 2 diabetes have diabetes secondary to pancreatic disease, which is frequently termed type 3c diabetes (including both PDAC-related diabetes and chronic pancreatitis-related diabetes). In their study, researchers showed that the combination of adiponectin and interleukin-1 receptor antagonist could stratify patients with new-onset type 2 diabetes into those with type 2 diabetes and those with the less prevalent PDAC-related diabetes, who are commonly misdiagnosed as having type 2 diabetes. Machine learning technologies could also help to identify high-risk subgroups and potentially make a population-based screening of PDAC feasible. In a study published in *PLOS ONE* in 2021, Ananya Malhotra and colleagues from London School of Hygiene & Tropical Medicine (UK) conducted a case-control study using prospectively-collected electronic health records from primary care data, which included all symptoms, status variables (ie, obesity, smoking, alcohol, diabetes, age, sex, social deprivation, and the number of consultations in the time window of interest). Based on these factors, researchers developed a machine learning approach that was able to identify patients who were at high-risk of developing pancreatic cancer up to 20 months before diagnosis. The algorithm could also be used alongside other non-invasive biomarkers and potentially increase the success rate of early diagnosis of PDAC.

Although only a few non-invasive biomarkers for early diagnosis of PDAC are near clinical application, researchers continue to search for new candidate biomarkers with better performance and applicability. A combination of different biomarkers could help to improve early diagnosis. Before implementation in clinical practice, these emerging biomarkers should be independently validated in larger cohorts, and their real-world diagnostic ability needs to be evaluated in prospective studies. *eBioMedicine*, as part of *The Lancet Discovery Science*, will continue to serve as a publishing platform for validated biomarker studies for human diseases, including pancreatic cancer.

